# Fungal spinal infections: a narrative review on diagnosis, treatment strategies, and collaborative management approaches

**DOI:** 10.3205/dgkh000624

**Published:** 2026-02-17

**Authors:** Anand Kumar Das, Mainak Sinha, Rijhul Lahariya, Saraj Kumar Singh, Sona Bhardwaj, Simmi Kishore

**Affiliations:** 1 MCh Neurosurgery, All India Institute of Medical Sciences, Patna, Bihar, India; 2 Department of General Surgery, All India Institute of Medical Sciences, Patna, Bihar, India; 3 MBBS, All India Institute of Medical Sciences, Patna, Bihar, India; 4 Department of Microbiology, ESIC Hospital, Patna, Bihar, India; 5 Department of Anaesthesiology and Critical Care Medicine, Indira Gandhi Institute of Medical Sciences, Patna, Bihar, India

**Keywords:** fungal infections, spine infections, vertebral osteomyelitis, spondylodiscitis, anti-fungal drugs

## Abstract

**Introduction:**

Spinal fungal infections are rare with a frequency of 2.2 times per 100,000 people annually. They are caused by pathogens such *Coccidioides immitis, Blastomyces dermatitidis, Cryptococcus neoformans, Candida albicans*, and *Aspergillus fumigatus* (found globally).

This review on fungal spinal infections examines patient demographics, medicinal treatments, surgical methods, and outcomes.

**Method:**

All PubMed articles on fungal spine infections were analysed, regardless of fungus, publication year, or spinal segment. The patient’s age, gender, affected spinal section, microorganism, treatment regimens, surgical methods, follow-up period, and results were recorded.

**Results:**

Of the 134 analyzed patients, 66.4% were male. The mean age was 54.3±14.9 years. Most susceptible was the lumbar spine (47%), followed by the thoracic (29.9%) and lumbo-sacral (12.7%). The most common organism was *Candida albicans* (62.7%), followed by *Aspergillus fumigatus* (27.6%). Spondylodiscitis (35.8%) and osteomyelitis (31.3%) lead our review.

The most frequent antifungals were triazoles (55%), followed by polyenes (31%). Most procedures (59.5%) were non-fixation.

Back discomfort, fever, and neurological impairments were prominent signs of spine fungal infections. Blood tests, cultures, and MRI/CT scans were used to diagnose.

Spondylodiscitis had a letality of 6.25%, spinal osteomyelitis of 11%. Recovery requires long-term monitoring.

**Conclusion:**

The review emphasises the significance of early detection and treatment, individualised antifungal regimens, and surgery in specific cases to enhance fungal spinal infection outcomes. Spine surgeons and radiologists must work together to solve diagnostic and therapeutic issues.

## Zusammenfassung

**Einleitung:**

Pilzinfektionen der Wirbelsäule sind selten mit einer jährlichen Häufigkeit von 2,2/100.000 Menschen. Sie werden durch Erreger wie *Coccidioides immitis, Blastomyces dermatitidis, Cryptococcus neoformans, Candida albicans* und *Aspergillus fumigatus* (weltweit verbreitet) verursacht.

Im vorliegenden Review über Pilzinfektionen der Wirbelsäule werden demografische Daten der Patienten, medikamentöse Behandlung, chirurgische Methoden und Therapieergebnisse analysiert.

**Methode:**

Es wurden alle PubMed-Artikel über Pilzinfektionen der Wirbelsäule analysiert, unabhängig vom Pilz, Publikationsjahr oder Wirbelsäulensegment. Erfasst wurden Alter, Geschlecht, betroffener Wirbelsäulenabschnitt, Mikroorganismus, Behandlungsschemata, chirurgische Methoden, Nachbeobachtungszeit und Therapieergebnisse.

**Ergebnisse:**

Von 134 Patienten waren 66,4% männlich. Das Durchschnittsalter betrug 54,3±14,9 Jahre. Am häufigsten war die Lendenwirbelsäule betroffen (47%), gefolgt von der Brustwirbelsäule (29,9%) und der Lumbosakralwirbelsäule (12,7%). Spondylodiszitis (35,8%) und Osteomyelitis (31,3%) stehen an der Spitze unserer Untersuchung. Der häufigste Erreger war *Candida albicans* (62,7%), gefolgt von *Aspergillus funigatus* (27,6%).

Die häufigsten verordneten Antimykotika waren Triazole (55%), gefolgt von Polyenen (31%). Die meisten Eingriffe (59,5%) erfolgten ohne Fixierung.

Rückenbeschwerden, Fieber und neurologische Beeinträchtigungen waren die häufigsten Anzeichen einer Pilzinfektion der Wirbelsäule. Zur Diagnose wurden Bluttests, Kulturen und MRT/CT-Scans verwendet.

Die Letalität betrug bei der Spondylodiszitis 6,25%, bei spinaler Osteomyelitis 11%. Die Genesung eine langfristige Überwachung erfordert.

**Schlussfolgerung:**

Die Ergebnisse unterstreichen die Bedeutung der frühzeitigen Erkennung und Behandlung, einer individuell abgestimmten antimykotischen Therapie und der Operation in bestimmten Fällen, um die Therapieergebnisse zu verbessern. Wirbelsäulenchirurgen und Radiologen müssen zusammenarbeiten, um diagnostische und therapeutische Probleme zu lösen.

## Introduction

Fungal infections of the spine are relatively uncommon. The complexity of diagnosing and treating spinal infections often demands a collaborative, cross-disciplinary approach involving spine surgeons and radiologists. The primary causes of spinal infections tend to be bacterial or fungal pathogens entering through the bloodstream, with *Staphylococcus aureus* and *Escherichia coli* being the most prevalent bacteria responsible for such infections [1].

Specific fungi like *Coccidioides immitis* and *Blastomyces dermatitidis* limited to certain geographical areas, while others like Cryptococcus spp., Candida spp., and Aspergillus spp. are found worldwide [2]. Coccidioidomycosis and Blastomycosis is prevalent in the American region [2]. Aspergillosis and Mucormycosis is more prevalent in the Asian region, especially in India [3].

The incidence rate of spinal infections is roughly 2.2 new cases annually per 100,000 individuals [4]. Spinal infections can be grouped according to the precise anatomical location impacted, including the vertebrae, intervertebral discs, spinal canal, and adjacent soft tissues [1]. The lumbar region is the area of the spine most commonly involved in spinal infections, followed by the thoracic and cervical regions, while hematogenous spread of infection to the sacral spine is relatively rare [4].

This is a comprehensive review of spinal infections caused by various fungi, presented according to the region in the spine affected, with patient’s demographic details, their medical therapy and surgical management, and follow-up along with their eventual outcome. This study explored studies from various parts of the world and is presented in this review article.

## Method

This review article has included articles published in National Library of Medicine (PubMed), using the search syntax ([mycotic OR fungal OR infection OR candida OR spondylodiscitis OR osteomyelitis] AND [spine OR spinal]). We included all fungal infections related to any segment of the spine. We tried to collect all relevant data from each of the articles including, age and gender of the patient, their spinal segment, the fungus involved, their follow-up duration, their medical therapy and surgical intervention, the main diagnosis of the patient and the overall outcome of the patient.

We included all patient above the age of 18 years, and made no restrictions on gender. We also made no restrictions related to any fungus, year of publication, follow-up duration or spinal segment. Articles in English literature only, from all over the world were included in this review. In medical therapy, we collected the details about the antifungal drug regimen used for the patient with the class of drug used. Under surgical intervention, we gathered the details about whether any intervention done or not. If yes, then was it without fixation or with fixation. Outcomes parameters included the follow-up duration of the patient, and their recovery and mortality status. Tab. 1 in the supplement (Attachment 1) shows all studies included in this review. 

## Results

The mean age of the included patients was 54.3 years±14.9 years. 89 were male (66.4%). The distribution of affected spinal regions varied, with the most common being lumbar (47%), followed by thoracic (29.9%), and lumbo-sacral (12.7%). Candida spp. were the most prevalent organisms identified, accounting for 62.7% of cases, followed by Aspergillus spp. (27.6%), with other organisms being relatively rare. Spondylodiscitis was the most frequent diagnosis (35.8%), followed by osteomyelitis (31.3%), and discitis (17.9%). Triazoles were most prescribed in 55% of cases, followed by polyenes (31%), and echinocandins (8.5%). The majority of interventions involved surgery, with 59.5% undergoing surgery without fixation and 40.5% undergoing surgery with fixation. Among these, the most common cause of surgery with fixation was discitis. Among the 131 individuals with reported outcomes, 90.1% were alive at the conclusion of the study, while 9.9% had died. The most common cause of death among the participants was spinal abscess (38.5%). The mean follow-up period (in months) for participants included in this review was 18.5 months±15 months (Table 1 (Tab. 1)). 

The fungal detection in various spine regions is summarised in Table 2 (Tab. 2). Only Aspergillus, Candida and Cryptococcus spp. were detected in different regions.

Table 3 (Tab. 3) is the summarised form of all the details about the participants we included, according to the diseases.

## Discussion

### Etiology

Candida and Aspergillus spp. are the most common causes of fungal vertebral osteomyelitis in immunocompromised patients [5]. Candida spp. were the most prevalent fungi, followed by Aspergillus, Cryptococcus, Histoplasma and Nakaseomyces spp. Candida and Aspergillus spp. were also the most common microorganisms for causing spinal cord abscess, a complication of osteomyelitis, caused most commonly by Candida spp. This finding was supported by other articles too [6], [7]. The most common microorganisms causing spondylodiscitis were found to be Candida and Aspergillus spp. in the reviewed articles. In rare cases Blastomyces, Paracoccidioides, Cryptococcus and Paecilomyces spp. causing spondylodiscitis. Among the discitis patients, the most common organisms involved were Candida and Aspergillus spp., few cases of Cryptococcus spp. and there was one case of Penicillium spp. infection. This was also supported by a few more studies [8], [9], [10].

### Presentation

From the articles reviewed, the mean age of vertebral osteomyelitis presentation was 53 years with a standard deviation of 14.5 years, with a male predominance, which corresponds with other studies [1], [11]. The most prevalent site affected was the lumbar spine, followed by the thoracic spine and the thoraco-lumbar region, supporting the current evidence [12]. The common symptoms that patients present with are back pain, fever, chills, muscle spasms, weight loss, and neurological deficits like numbness and weakness, like reported in the literature [1]. Spinal cord abscess is more prevalent in the population of males over 52.8 years with a standard deviation of 20 years [1], [13]. Thoracic spine was the most frequently affected, followed by the lumbar and lumbo-sacral regions. The common symptoms that patients present with spinal abscess include fever, chills, loss of bladder and bowel control, loss of sensation in the body area below the abscess, headache, and muscle weakness, especially in the legs [6], [14], [15]. In spondylodiscitis, males are affected more, in the age group of 55.5 years with a standard deviation of 14.1 years, analogous as reported of Yu et al. [16]. In accordance with the literature [16], [17], the most common site affected by spondylodiscitis was the lumbar spine, followed by the thoracic spine and the lumbo-sacral region. The most common manifestation was lower back pain, with other features like fever, chills, some neurological manifestations, an abscess, and sometimes vertebral collapse, which corresponds with the literature [17], [18], [19]. Discitis has a higher incidence among the 50-year-old age group, with male predominance as also reported in the literature [10]. 

The mean age of the patients who had discitis was 54.5 years (SD 14.7 years) with male percentage of 62.5%. 

The lumbar region was to be most affected, followed by the lumbo-sacral and thoracic regions, which corresponds with the literature [9]. In accordance to the literature [20], [21], [22], the common signs and symptoms of discitis include non-relieving back pain or neck pain, fever, weight loss, motor weakness that eventually causes paralysis, abdominal discomfort, and sensory impairment.

### Diagnosis

The diagnosis of vertebral osteomyelitis is somewhat dependent on several blood tests and imaging techniques. Complete blood count, erythrocyte sedimentation rate, and C-reactive protein are reliable indicators of inflammation [1], [23]. Blood culture is done to find out the organism involved along with its species for effective antibiotic treatment and intervention [23]. Imaging tools are available that are effective in making a diagnosis. A computed tomography (CT) scan is best to diagnose the structural deformity of the bone. Magnetic Resonance Imaging (MRI) is considered the gold standard as it can view infected tissues like muscles, nerves involved, and other soft tissues [1]. Gallium-67 single-photon emission computed tomography (SPECT) was being used as an alternative to MRI with sensitivity comparable to MRI in patients where MRI was contraindicated [23], [24]. And if the doubt persists, a biopsy is done using CT-guided aspiration or biopsy. MRI is also helpful in guiding the site for CT-guided tissue biopsy [23].

The diagnosis of spinal cord abscess is generally made by considering inflammatory markers like white blood cell count (WBC), erythrocyte sedimentation rate (ESR), and c-reactive protein (CRP), which will be raised [6], [13], [15]. Culture can be done with the pus and/or blood sample to find the causative organism for effective treatment. Among various imaging techniques, MRI with gadolinium is the most reliable technique to detect spinal abscesses with sensitivity and specificity above 90% [13], [15]. It also aids in the detection of vertebral osteomyelitis, which is the primary risk factor for spinal cord abscess. Other techniques, like CT with intravenous contrast media, are considered an alternative choice for diagnosis [6], [13], [15]. 

The mainstay of the treatment remains antibiotic therapy, but the surgeon needs to drain the abscess with a needle to release the pressure [15]. Other procedures, like laminectomy and corpectomy, can be employed to drain the abscess [6]. In many of the cases, the culture may give a false negative result, so early CT-guided needle aspiration of the abscess is to be done [13].

The diagnosis of spondylodiscitis depends on laboratory and imaging techniques. Routine inflammation markers, including ESR and CRP, are usually a bit elevated. MRI stands out as the most sensitive imaging technique for making the diagnosis. Thorough investigations related to the microbiological tests should be done to confirm the infective organisms for effective antifungal therapy. A CT-guided biopsy and fine needle aspiration cytology are done to confirm the diagnosis and for proper management of the condition [16], [19]. Delayed diagnosis and initiation of antifungals would lead to a poor outcome [19].

The first step in diagnosing discitis is to check for the infection through inflammatory markers like ESR and CRP that are likely to be raised [21]. A positive blood culture is needed to find the causative organism, whether it’s fungal, bacterial, or parasitic, to start the correct antibiotic regime [21]. MRI is considered to be the most sensitive and better diagnostic technique, but the gold standard approach is the CT-guided tissue biopsy [10], [21].

### Management

The mainstay treatment for vertebral osteomyelitis is the antifungal therapy, along with the immobilisation for increased time in the patient with severe back pain and spinal deformity, followed by rehabilitation exercises for muscle strengthening [1], [23]. In our review, the most commonly used antifungal was triazoles like voriconazole, fluconazole, and itraconazole, followed by polyenes like nystatin, amphotericin B, liposomal preparation of amphotericin B and few cases with antimetabolite group of drugs. Non-surgical intervention is considered primarily if no severe complications present or no severe symptoms develop in the patient, like a neurological deficit [1]. Surgical intervention has higher mortality rates and includes either debridement of the tissue that is infected or the use of instrumentation to fix the spine to gain stability [1]. Still, many of the patients have poor quality of life as the recovery is prolonged [23]. In our review, the majority of the cases were managed with some intervention without any fixation and just debridement of the infected tissue, with a single case of management with fixation of the spine. 

For spinal cord abscess treatment, azoles and polyene groups of drugs were the mainstays of antifungal therapy. The majority of the cases reported were managed with surgical intervention but without any fixation; just laminectomy, corpectomy, and decompression for drainage of the abscess were the primary procedures. 

Among the antifungals for the treatment of spondylodiscitis, amphotericin B and azoles are considered the primary antifungal therapies. In our review, azole were used in the majority of the cases, followed by polyenes and echinocandine, which were equally used. If neurological symptoms persist or vertebral collapse is present, only then are surgical interventions considered [19]. Around 80% of the cases were managed with slight intervention but without any fixation. Early decompression enhances the functional recovery of most patients. Just debridement and draining the abscess treat the patient, along with stabilising the spine, as summarized by Caldera et al. [19].

The main antifungal therapies advised to the patient in our review were triazoles and polyenes, including voriconazole, fluconazole, itraconazole and amphotericin B respectively. Surgical intervention is indicated only when some neurological deficits are observed, such as abscess formation that needs to be drained, vertebral cord prolapse, or chronic illness. Decompression for drainage of the abscess was done, and surgical debridement was done for effective medical therapy. In our review, all cases reported for discitis were managed with surgical intervention with fixation of the spinal cord. After fixation, timely follow-up is very much required. 

### Prognosis

In case of vertebral osteomyelitis, timely follow-up is very necessary for the patient to make a functional recovery as soon as possible. The mean follow-up time for the patient was 20.3 months (SD 16.4 months). And because of that, the mortality rate among the articles we reviewed for vertebral osteomyelitis was around 11%. The common causes of death among patients of osteomyelitis were progression to sepsis, which is a life threatening condition. Patients with compromised immune systems were also at great risk [4]. Among the spinal cord abscess patients, more than half of the cases were reported as alive, with a mean follow-up time of 9.63 months (SD 8.83 months). In case of spondylodiscitis, the mean follow-up time was found to be 11.3 months (SD 9.03 months). And because the majority of the patients were managed with minor procedures and without any fixation, the mortality rate was found to be only 6.25% in patients with spondylodiscitis. Short-term mortality is associated with severe neurologic deficits, epidural abscess, and comorbidities. Long term mortality is related to alcohol dependency [25]. Among the discitis patients, the mean time of follow-up was 34.2 months (SD 11.3 months). After fixing the cord, there was zero mortality rate among the patients reported in our review.

## Conclusion

Fungal infections involving the spinal column, comprises of vertebral osteomyelitis, spinal cord abscess formation, spondylodiscitis, and discitis, present significant diagnostic hinderance that are frequently overlooked due to their infrequent occurrence and nonspecific symptoms. Timely diagnosis necessitates a synergistic approach, combining laboratory analysis and advanced imaging techniques, with MRI emerging as the diagnostic gold standard. Effective management of these conditions requires a multidisciplinary treatment strategy, integrating antifungal pharmacotherapy and surgical interventions when deemed appropriate. Despite the intricacies associated with the management of these conditions, timely intervention and a comprehensive care approach contribute to favorable clinical outcomes, underscoring the paramount importance of heightened clinical vigilance and collaborative interdisciplinary practice in addressing these rare yet consequential pathologies.

## Notes

### Authors’ ORCIDs

- Das AK: 0000-0002-0705-9393
- Sinha M: 0000-0002-2286-0701
- Lahariya R: 0009-0003-5769-4509
- Singh SK: 0000-0003-3156-7096

### Contribution

Authors Anand Kumar Das and Mainak Sinha contributed equally.

### Funding

None. 

### Acknowledgments

The authors acknowledge all professors and consultants in the department of Neurosurgery for their guidance and assistance.

### Competing interests

The authors declare that they have no competing interests.

## Supplementary Material

Table 1: All studies included in the review

## Figures and Tables

**Table 1:**
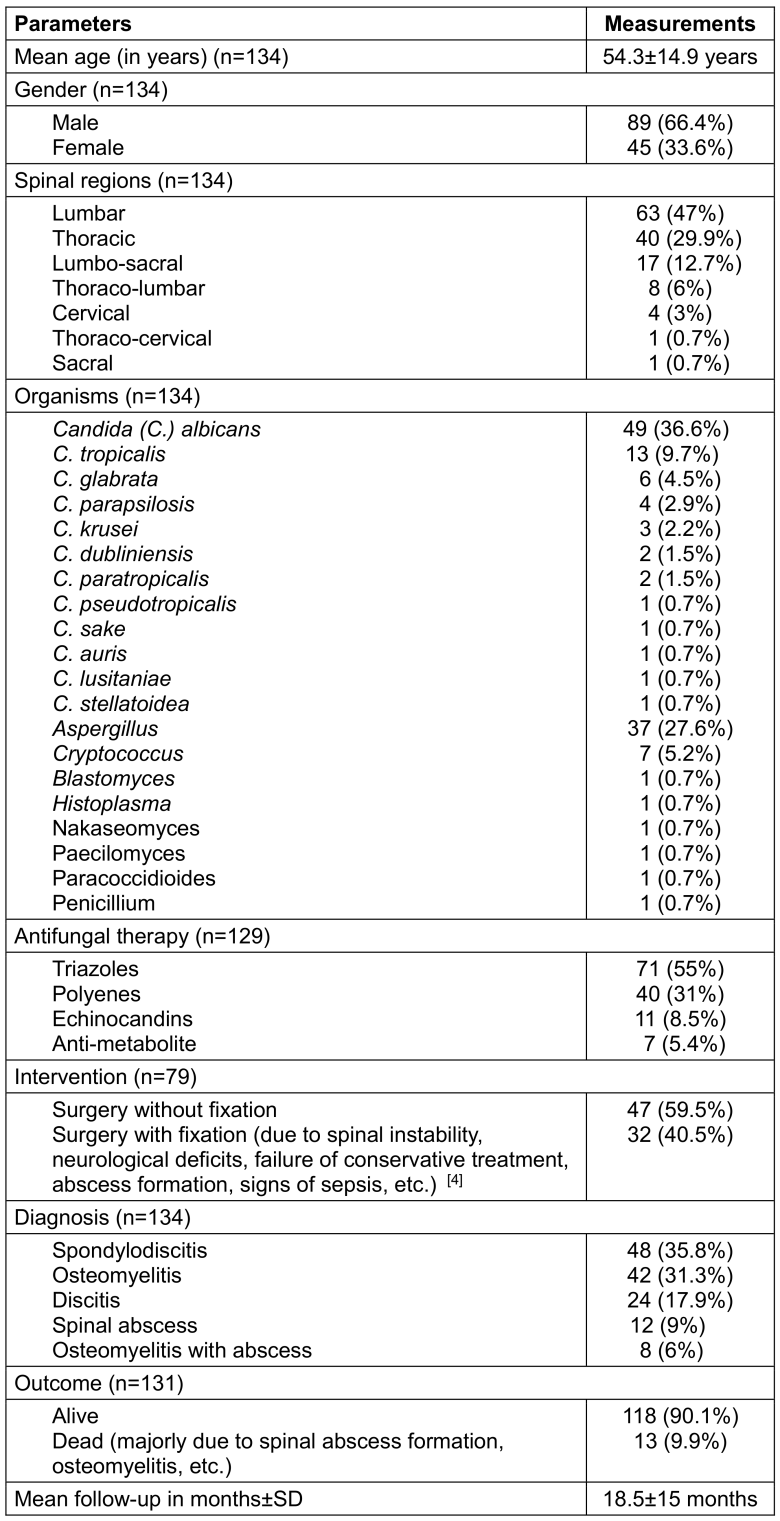
Demographics of the patient population, affected spine region, isolated fungi and therapy

**Table 2:**
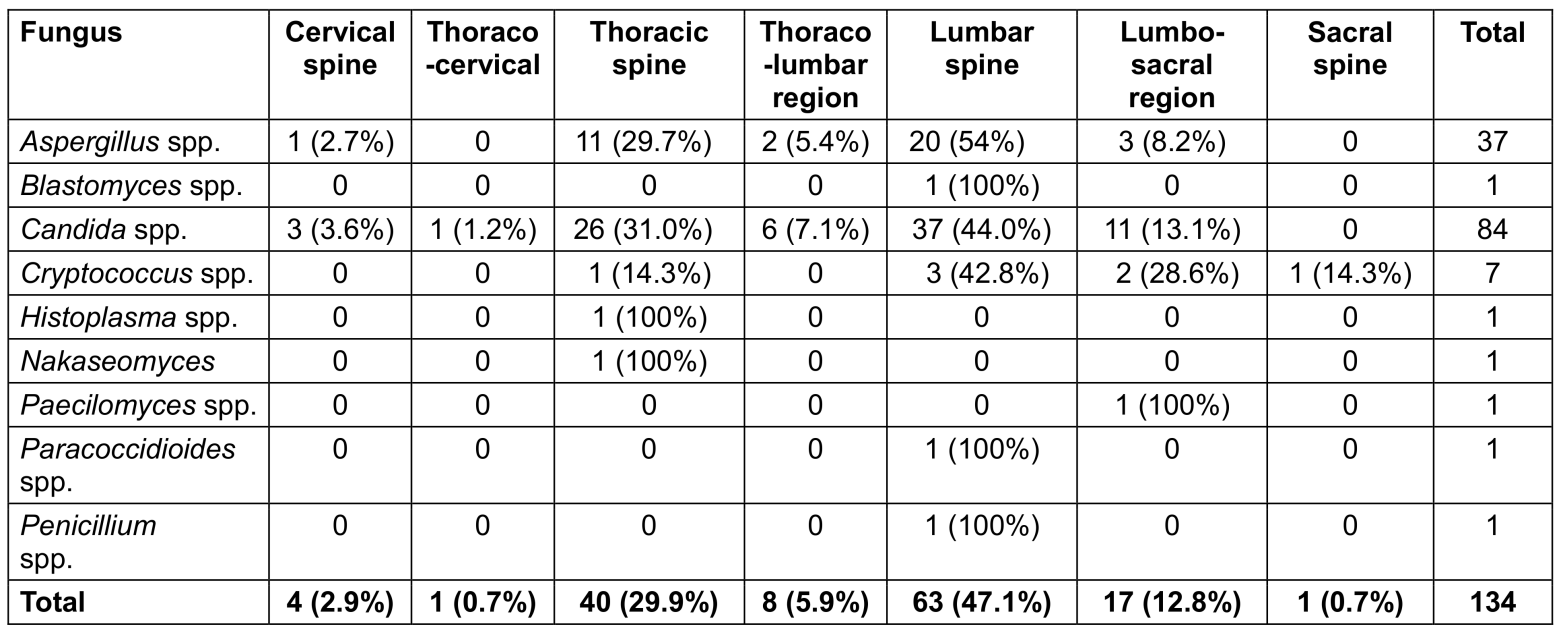
Distribution of different fungi within the spine

**Table 3:**
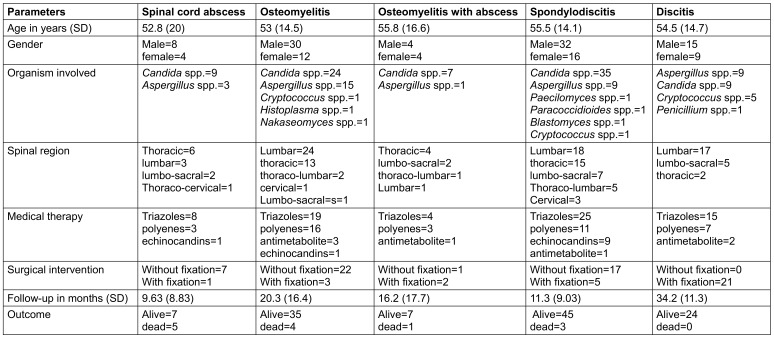
Detailed review of all articles on the basis of the diagnosis
