# Supplementary material for: Fungal spinal infections: a narrative review on diagnosis, treatment strategies, and collaborative management approaches
Source: GMS Hyg Infect Control. 2026 Feb 17;21:Doc15. doi: 10.3205/dgkh000624 (PMC12973500; doi:10.3205/dgkh000624)
Supplement: Table 1: All studies included in the review [file HIC-21-15-s-001.pdf]

Table 1: All studies included in the review

| Sl no | Author | Age | Sex | Spinal level | Microrganism | No of patient | Follow up [months] | Medical therapy | Surgical intervention | Diagnosis | Outcome |
|---|---|---|---|---|---|---|---|---|---|---|---|
| 1 | Son et al. 2007 [26] | 46 | M | C4-5 | Aspergillus (A.) spp. | 1 | | | | Tuberculous spondylitis | |
| | | | | L3-5 | | | | | | | |
| | | | | T2-4 | | | | | | | |
| 2 | D'Agostino et al. 2010 [27] | 66 | F | C6-7 | *Candida (C.) glabrata* | 6 | 2.75 | Amphotericin B | | Infectious spondylodiscitis | A |
| | | 74 | M | L1-2 | *C. albicans* | | 2 | Fluconazole | Spinal surgery | | A |
| | | 53 | M | L2-3 | *C. albicans* | | 2.75 | Fluconazole and amphotericin B | | | A |
| | | 60 | M | L4-5 | *A. fumigatus* | | 6.5 | Voriconazole | | | A |
| | | 61 | M | L4-5 | Aspergillus spp. | | 7.5 | Voriconazole | | | A |
| | | 60 | F | T12-L4 | *C. albicans* | | 8.25 | Fluconazole | | | A |
| 3 | Huang et al. 2019 [28] | 64 | F | C4-6 | *C. albicans* | 1 | | Fluconazole | Corpectomy of C4, C5, C6 with radial resection of tissues | Infective spondylodiscitis at C5-6 | A |
| 4 | El Khoury et al. 2018 [29] | 53 | F | C4-5 | *C. glabrata* | 1 | 3 | Micafungin and voriconazole | | Spondylodiscitis | A |
| 5 | Raj et al. 2013 [6] | 45 | F | L5-S1 | *A. fumigatus* | 1 | 9 | Itraconazole | Posterior decompression, laminectomy | Paradiscal infective spondylodiscitis | A |
| 6 | Yang et al. 2019 [30] | 74 | M | L3-S1 | *C.albicans* | 9 | 0.5 | Caspofungin, flucytosine, fluconazole | | Fungal spinal epidural abscess | D |
| | | 40 | M | L5-S1 | *C. albicans* | | 19.25 | Fluconazole | Microdiscectomy | | A |
| | | 77 | M | L3-4 | *C. glabrata* | | | Micafungin and fluconazole | Laminectomy [posterior] | | D |
| | | 21 | F | L3-4 | *C. albicans* | | 7.25 | Fluconazole and caspofungin | Debridement and Drainage | | A |
| | | 54 | M | T11-12 | *C. albicans* | | 10.75 | Amphotericin B, nafcillin, fluconazole | | | A |
| | | 68 | M | T11-12 | *C. albicans* | | 2.5 | Flucytosine, micafungin, fluconazole | | | D |
| | | 48 | F | T3-5 | *A. fumigatus* | | 2.25 | Amphotericin B and voriconazole | Laminectomy, corpectomy and instrumentation | | A |
| | | 51 | M | T5-10 | *A. fumigatus* | | | Amphotericin B | Laminectomy | | D |
| | | 63 | F | C6-T1 | *C. albicans* | | 25.5 | Amphotericin B | Laminectomy, corpectomy and iliac crest bone fusion | | A |
| 7 | Boyd et al. 2018 [31] | 71 | F | L5-S1 | *C. albicans* | 1 | 12 | Fluconazole | | Vertebral osteomyelitis | A |
| 8 | Kroot and Wouters 2007 [32] | 28 | M | L5-S1 | *C. albicans* | 1 | | Fluconazole | | L5–S1 Spondylodiscitis | A |
| 9 | Iwata et al. 2014 [18] | 55 | M | L5-S1 | *Paecilomyces variotii* | 4 | 26 | Amphotericin B and itraconazole | Discectomy | Disc herniation and spondylodiscitis | A |

Attachment 1 to: Das AK, Sinha M, Lahariya R, Singh SK, Bhardwaj S, Kishore S. Fungal spinal infections: A narrative review on diagnosis, treatment strategies, and collaborative management approaches. GMS Hyg Infect Control. 2025;20:Doc15. DOI: 10.3205/dgkh000624

| | | | | | | | | | | | |
|---|---|---|---|---|---|---|---|---|---|---|---|
| | | 56 | M | L3-4 | *C. albicans* | | 92 | Fluconazole and amphotericin B | Posterolateral endoscopic debridement and irrigation | Spinal infection | A |
| | | 72 | M | L3-4 | *C. albicans* | | 24 | Micafungin | Bi-portal approach | | |
| | | 56 | M | L2-3 | *C. albicans* | | 28 | Voriconazole | Posterolateral endoscopic debridement and irrigation | | |
| 10 | Oksi et al. 2013 [12] | 37 | M | L5-S1 | *C. dubliniensis* | 1 | 3 | Amphotericin B and fluconazole | | Spondylodiscitis | A |
| 11 | Grimes et al. 2012 [33] | 63 | F | L5-S1 | *C. albicans* | 1 | 3 | Fluconazole and Mmicafungin | L4-5 discectomy and L5 corpectomy | Osteomyelitis and discitis | A |
| 12 | Salzer et al. 2015 [34] | 47 | M | L4-S1 | *C. dubliniensis* | 1 | | Fluconazole | | Spondylodiscitis and spinal abscess | A |
| 13 | de Matos et al. 1998 [35] | 45 | M | L5-S1 | *C. albicans* | 1 | | Fluconazole | | L5/S1 Spondylodiscitis | A |
| 14 | Yu et al. 2016 [16] | 76 | M | L5-S1 | *C. albicans* | 1 | 12 | Fluconazole | Auto-bone graft and interbody fixation | Spondylodiscitis | A |
| 15 | Jorge et al. 2012 [36] | 75 | M | L5-S1 | *C. albicans* | 1 | 6 | Fluconazole and amphotericin B | | L5/S1 Spondylodiscitis with a spinal epidural abscess | A |
| 16 | Zou et al. 2015 [37] | 56 | M | L5-S1 | *A. fumigatus* | 15 | 51 | Amphotericin B and itraconazole | Anterior radical debridement and interbody fusion | Fungal discitis | A |
| | | 31 | F | | *A. fumigatus* | | 30 | voriconazole | | | |
| | | 32 | F | | *Cryptococcus (Cr.) neoformans* | | 28 | amphotericin B | | | |
| | | 57 | F | | *Cr. neoformans* | | 36 | Fluconazole | | | |
| | | 59 | M | | *C.albicans* | | 36 | Amphotericin B | | | |
| | | 56 | M | L3-5 | *A. fumigatus* | | 51 | Amphotericin B and itraconazole | | | |
| | | 31 | F | L3-5 | *A. fumigatus* | | 30 | Voriconazole | | | |
| | | 42 | M | L4-5 | *A. fumigatus* | | 45 | Voriconazole | Anterior radical debridement, interbody fusion and posterior instrumented fusion | | |
| | | 62 | M | L3-4 | *A. fumigatus* | | 24 | Amphotericin B and itraconazole | | | |
| | | 32 | F | L3-5 | *Cr. neoformans* | | 28 | Amphotericin B | Anterior radical debridement and interbody fusion | | |
| | | 57 | F | L3-5 | *Cr. neoformans* | | 36 | Fluconazole | | | |
| | | 48 | M | L3-4 | *Cr. neoformans* | | 48 | Fluconazole | | | |
| | | 61 | M | L3-5 | *C. albicans* | | 32 | Amphotericin B | Anterior radical debridement, interbody fusion and posterior instrumented fusion | | |
| | | 56 | F | L3-4 | *C. albicans* | | 30 | Amphotericin B | Anterior radical debridement and interbody fusion | | |
| | | 59 | M | L3-5 | *C. albicans* | | 36 | Amphotericin B | | | |

| | | | | | | | | | | | |
|---|---|---|---|---|---|---|---|---|---|---|---|
| 17 | Zhong et al. 2023 [38] | 37 | M | S2-4 | *Cr. neoformans* | 1 | 8 | Fluconazole | Sacral debridement and negative drainage | Cryptococcal osteomyelitis of the sacrum | A |
| 18 | Ur-Rahman et al. 2000 [39] | 40 | F | T6-8 | Aspergillus spp. | 1 | | Amphotericin B | | Osteomyelitis | D |
| 19 | Vaishya and Sharma 2004 [40] | 35 | F | T10-12 | Aspergillus spp. | 1 | | Amphotericin B | T11 Corpectomy | Vertebral osteomyelitis with extradural abscess | D |
| 20 | Sethi et al. 2012 [41] | 25 | M | L4-5 | Aspergillus spp. | 2 | 12 | Itraconazole | Posterior decompression and interbody fusion with tricortical bone graft from the iliac crest | Fungal osteomyelitis | A |
| | | 19 | M | T10-11 | Aspergillus spp. | | | Itraconazole | Transthoracic T10-T11 corpectomy | Osteomyelitis | A |
| 21 | Eisen et al. 2000 [42] | 73 | M | T8-9 | *C. tropicalis* | 1 | 9 | Amphotericin B | Surgical decompression | Vertebral osteomyelitis | A |
| 22 | Derkinderen et al. 2000 [43] | 32 | F | T6-9 | *C. albicans* | 1 | 11 | Amphotericin B and flucytosine | | Candidal spondylodiscitis and epidural abscess | A |
| 23 | Sebastiani and Galas 2001 [44] | 67 | M | T6-9 | *C. tropicalis* | 1 | | Fluconazole | | Spondylodiscitis | A |
| 24 | Rachapalli et al. 2010 [45] | 48 | F | T6-7 | Candida spp. | 1 | | Fluconazole and flucytosine | | Discitis | A |
| 25 | Rossel et al. 1998 [46] | 20 | F | T11-12 | *C. albicans* | 1 | 12 | Fluconazole | | Spondylitis/discitis | A |
| 26 | Peman et al. 2006 [47] | 62 | M | T5-6 | *C. krusei* | 1 | 6 | Caspofungin and voriconazole | | Spondylodiscitis | D |
| 27 | Saigal et al. 2004 [7] | 31 | F | L3-5 | *A. fumigatus* | 1 | | Amphotericin B | T9-T11 Laminectomy | Discitis and osteomyelitis | A |
| | | | | T9-11 | *A. fumigatus* | | | Amphotericin B | T9-T11 Laminectomy | Intradural abscess | |
| 28 | Jiang et al. 2013 [48] | 40 | F | T1-3 | *A. nidulans* | 1 | 16 | Voriconazole | Decompression laminectomy of T1-T3 | Osteomyelitis | A |
| 29 | Chia et al. 2005 [49] | 50 | M | T7-8 | *C. tropicalis* | 1 | 3 | Amphotericin B | Anterior decompression | Spondylodiscitis at T7/T8 associated with osteomyelitis of T7 and T8 vertebrae | A |
| 30 | Ackerman and Bayley 1990 [50] | 66 | M | T6-7 | *C. albicans* | 1 | | Amphotericin B | | Spondylodiscitis | A |
| 31 | | 45 | M | T4-5 | Aspergillus spp. | 3 | | Amphotericin B | | Spondylodiscitis of T4-T5 | A |

Attachment 1 to: Das AK, Sinha M, Lahariya R, Singh SK, Bhardwaj S, Kishore S. Fungal spinal infections: A narrative review on diagnosis, treatment strategies, and collaborative management approaches. GMS Hyg Infect Control. 2025;20:Doc15. DOI: 10.3205/dgkh000624

| | | | | | | | | | | | |
|---|---|---|---|---|---|---|---|---|---|---|---|
| | van Ooij et al. 2000 [51] | 39 | F | L4-5 | *A. fumigatus* | | | Amphotericin B | Lumbotomy | AML | D |
| | | 69 | M | T12-L1 | *A. fumigatus* | | | Amphotericin B and flucytosine | Decompression | Spondylodiscitis at T12-L1 | D |
| 32 | Ugarriza et al. 2004 [52] | 70 | M | T8-9 | *C. albicans* | 1 | 4 | Fluconazole | Curettage and debridment followed by grafting with autologous bone | T8 –T9 Spondylodiscitis | A |
| 33 | El-Zaatari et al. 2002 [53] | 65 | M | T11-12 | *C. albicans* | 1 | 12 | Fluconazole | Surgical debridement, fixation, and Bone grafting of the lesion | Osteomyelitis of the spine | A |
| 34 | Friedman and Simon 1987 [54] | 77 | M | T9-10 | *C. paratropicalis* | 3 | 24 | Amphotericin B | | Discitis and osteomyelitis | A |
| | | 79 | M | L4-5 | *C. pseudotropicalis* | | 24 | Amphotericin B | | Vertebral osteomyelitis | A |
| | | 44 | M | L4-5 | *C. parapsilosis* | | 18 | Amphotericin B | | Vertebral osteomyelitis | A |
| 35 | Torres-Ramos et al. 2004 [55] | 69 | F | T8-9 | *C. tropicalis* | 1 | | Amphotericin B | | Spondylodiscitis | A |
| 36 | Pennisi et al. 1985 [56] | 77 | M | T9-10 | *C.paratropicalis* | 2 | | Amphotericin B | Right costo-transversectomy of T10 with luqus rod insertion | Diskitis | A |
| | | 79 | M | L3-4 | *C. tropicalis* | | | Amphotericin B and flucytosine | | Diskitis | A |
| 37 | Hennequin et al. 1996 [57] | 52 | F | T10-11 | *C.. albicans* | 2 | 47 | Fluconazole | | Spondylodiscitis with abscess | A |
| | | 61 | M | L3-4 | *C. albicans* | | 17 | Fluconazole | | Spondylodiscitis | A |
| 38 | Mullins et al. 1993 [58] | 38 | M | T8-9 | *C. albicans* | 1 | | Amphotericin B | | Osteomyelitis | A |
| 39 | Crane 2018 [59] | 27 | M | T3-8 | *C. albicans* | 1 | | Fluconazole | Laminectomy of T6–T8 | Intrathecal spinal abscesses | A |
| 40 | Kashimoto et al. 1986 [60] | 50 | M | T7-8 | *C. tropicalis* | 1 | 21 | | T6-7 Thoracotomy | Vertebral osteomyelitis and discitis | A |
| 41 | Liu et al. 2017 [61] | 65 | M | T4-6 | Histoplasma spp. | 1 | 26 | Itraconazole | Thoracic spinal surgery | Vertebral histoplasmosis | A |
| 42 | Lee et al. 2017 [62] | 66 | F | T10-11 | *C. albicans* | 1 | | Fluconazole | Open curettage and spinal stabilization | Spondylodiscitis with abscess | A |
| 43 | Kelesidis and Tsiodras 2012 [63] | 41 | M | T11-12 | *C. albicans* | 1 | 10 | Caspofungin | | Spondylodiscitis | A |
| 44 | Bruns et al. 1986 [64] | 63 | F | T7-8 | *C. tropicalis and Nakaseomyces glabratus* | 1 | 18 | Amphotericin B and flucytosine | Costotransversectomy | Spondylitis | A |
| 45 | Kulcheski et al. 2015 [65] | 39 | M | T4-6 | *C. albicans* | 1 | 12 | Fluconazole and ciprofloxacin | Corpectomy from T4-6 with autologous iliac graft replacement | Thoracic spondylodiscitis | A |

| | | | | | | | | | | | |
|---|---|---|---|---|---|---|---|---|---|---|---|
| 46 | Neale et al. 1987 [66] | 36 | F | T10-11 | *C. albicans* | 1 | 6 | Flucytosine and amphotericin B | | Vertebral osteomyelitis | A |
| 47 | Relvas-Silva et al. 2020 [67] | 66 | F | T8-10 | *C. albicans* | 1 | 18 | Amphotericin B and anidulafungin | Transpedicular screw fixation between T5-T6 and T11-T12 for stability and anterior thoracic corpectomy of T9 with rib bone grafting | Spondylodiscitis | A |
| 48 | Takagi et al. 2019 [68] | 74 | M | T11-12 | *A. terreus* | 1 | 24 | Voriconazole | Posterior partial laminectomy at T11 and anterior fusion at T11 and T12 | Spondylodiscitis | A |
| 49 | Bhat et al. 2021 [69] | 52 | M | T10-12 | *Cr. neoformans* | 1 | | Amphotericin and fluconazole | | Pyogenic spondylodiscitis | A |
| 50 | Edwards et al. 1975 [70] | 74 | F | T10-11 | *C. albicans* | 2 | | Amphotericin B | | Osteomyelitis | D |
| | | 56 | M | T9-10 | | | | Flucytosine | Spinal fusion | | A |
| 51 | Dai et al. 2020 [71] | 67 | M | T3-5 | *A. fumigatus* | 6 | 20 | Voriconazole | | Aspergillus spondylitis | A |
| | | 50 | F | L3-4 | *A. fumigatus* | | 24 | Voriconazole | Laminectomy, debridement, instrumentation | | A |
| | | 48 | M | L4-5 | *A. fumigatus* | | 15 | Voriconazole | Laminectomy, debridement, instrumentation | | A |
| | | 43 | M | L4-5 | *A. niger* | | 20 | Voriconazole | Laminectomy, debridement, instrumentation | | A |
| | | 66 | M | L2-3 | Aspergillus spp. | | 18 | Voriconazole | Laminectomy, debridement, instrumentation | | A |
| | | 68 | M | T12-L2 | *A. fumigatus* | | 24 | Voriconazole | Laminectomy, debridement, decompression, instrumentation | | A |
| 52 | Tokuyama et al. 2002 [5] | 44 | F | T12-L1 | *C. albicans* | 1 | | Itraconazole | Total corpectomy of T12 and L1 | Spondylodiscitis | A |
| 53 | Williams et al. 1999 [72] | 51 | M | T12-L1 | Candida spp. | 3 | | Amphotericin and fFluconazole | | Candida osteomyelitis | A |
| | | 49 | M | L2-4 | *C. albicans* | | | Amphotericin | L2–L3 and L3–L4 Diskectomy and vertebral debridement | Fungal spinal osteomyelitis | A |
| | | 54 | F | L2-4 | Aspergillus spp. | | | Amphotericin | | | A |
| 54 | Lopes et al. 2021 [73] | 72 | M | T9-L4 | *C. tropicalis* | 1 | | Micafungin | T9-L4 Laminectomy, arthrodesis and abscess drain | Thoracolumbar spondylodiscitis | D |
| 55 | Er and Yilmaz 2020 [74] | 54 | F | T12-L1 | *C. parapsilosis* | 1 | | Fluconazole | | Spondylodiscitis | A |

| | | | | | | | | | | | |
|---|---|---|---|---|---|---|---|---|---|---|---|
| 56 | Sugar et al. 1990 [75] | 70 | M | T12-L1 | *C. tropicalis* | 1 | 14 | Amphotericin B and fluconazole | Rod stabilization of the vertebral column | Vertebral osteomyelitis with epidural abscess | A |
| 57 | Rashid et al. 2022 [76] | 58 | F | L4,5 | Aspergillus spp. | 1 | | Voriconazole and micafungin | | Foraminal stenosis | A |
| 58 | Yoon and Kim 2015 [77] | 53 | M | L2-3 | Aspergillus spp. | 1 | 7 | Vancomycin and amphotericin B | Total laminectomy of L2 | Infectious spondylodiskitis | A |
| 59 | Upadhyay et al. 2020 [78] | 45 | M | L4-5 | *A. flavus* | 5 | | Voriconazole | MI-PLIF | Postoperative fungal discitis | A |
| | | 59 | M | L4-5 | *A. flavus* | | | Voriconazole | | | A |
| | | 51 | M | L3-4 | *A. flavus* | | | Voriconazole | | | A |
| | | 61 | M | L4-5 | *C. albicans* | | | Amphotericin B and itraconazole | | | A |
| | | 60 | F | L4-5 | *Penicillium decumbens* | | | Amphotericin B and itraconazole | | | A |
| 60 | Seravalli et al. 2003 [79] | 64 | M | L2-4 | *C. glabrata* | 1 | 16 | Amphotericin B | Corpectomy of L2-L3 | vertebral osteomyelitis | A |
| 61 | Schilling et al. 2008 [80] | 58 | M | L2-3 | *C. krusei* | 1 | 12 | Caspofungin | | Progressive spondylodiscitis | A |
| 62 | Oichi et al. 2015 [81] | 79 | M | L3-4 | *C. tropicalis* | 1 | 12 | Micafungin and fluconazole | | Intervertebral abscess | D |
| 63 | Herzog et al. 1989 [82] | 88 | M | L4-5 | *C. tropicalis* | 1 | 6 | Amphotericin B and ketoconazole | | Intervertebral diskitis | A |
| 64 | Shaikh et al. 1980 [83] | 67 | M | L1-2 | *C. albicans* | 1 | | Amphotericin B and flucytosine | | Osteomyelitis | D |
| 65 | Hadjipavlou et al. 1998 [84] | 34 | F | L2-4 | *Blastomyces dermatitides* | 1 | 7 | Fluconazole and amphotericin B | Total excision of the second lumbar vertebral body | Disseminated blastomycosis | A |
| 66 | Savall et al. 2014 [85] | 22 | M | L1-2 | *C. albicans* | 1 | | Fluconazole | | Spondylodiscitis | A |
| 67 | Palmisano et al. 2011 [8] | 48 | F | L3-4 | *C. sake* | 1 | 6 | Fluconazole | | Spondylodiscitis | A |
| 68 | Duplan et al. 2022 [86] | 50 | M | L2-3 | *C. parapsilosis* | 1 | 12 | Fluconazole | L2 and L3 Laminectomy | Osteomyelitis | A |
| 69 | Chen and Lin 2012 [9] | 59 | M | L3-5 | *C. parapsilosis* | 1 | 12 | Fluconazole | L2-L5 Laminectomy | Discitis and osteomyelitis | A |
| 70 | Werner et al. 2011 [87] | 40 | F | L3-4 | *C. lusitaniae* | 1 | 24 | Fluconazole and amphotericin B | Left L3 and L4 hemilaminectomy with L3–L4 discectomy | Osteomyelitis | A |

| | | | | | | | | | | | |
|---|---|---|---|---|---|---|---|---|---|---|---|
| 71 | Dailey and Young 2011 [88] | 69 | M | L1-2 | *C. glabrata* | 1 | 3 | Amphotericin B | | Discitis, Osteomyelitis and epidural abscess | A |
| 72 | Fogarty 1983 [89] | 40 | F | L1-2 | *C. albicans* | 1 | 36 | Amphotericin B | | Osteomyelitis | A |
| 73 | Holzman and Bishko 1971 [90] | 41 | F | L4-5 | *C. stellatoidea* | 1 | | Amphotericin B | | Spondylitis | A |
| 74 | Chen et al. 2013 [91] | 41 | M | L3-4 | *C. albicans* | 1 | 9 | Fluconazole | Left laminotomy over L3 and discectomy over L3–4 | Spondylosis of lumbar spine | A |
| 75 | Rieneck et al. 1996 [92] | 56 | M | L3-4 | *C. albicans* | 1 | | Fluconazole | | Spondylitis | A |
| 76 | Gagliano et al. 2018 [93] | 66 | M | L3-4 | *C. glabrata* | 1 | | Anidulafungin | Debridement and stabilization of the vertebrae | Spondylodiscitis | A |
| 77 | Hayes et al. 1984 [94] | 67 | M | L1-2 | *C. tropicalis* | 1 | | | | Discitis and Vertebral osteomyelitis | A |
| 78 | Alvarenga et al. 2016 [95] | 68 | M | L4-5 | *Paracoccidioides brasiliensis* | 1 | 36 | Sulfamethoxazole and trimethoprim | | Spondylodiscitis | A |
| 79 | Munk et al. 1997 [96] | 67 | M | L2-3 | *C. albicans* | 1 | | Amphotericin B | Retroperitoneal vertebral body resection, abscess drainage, and strut grafting | Osteomyelitis and disc space infection | A |
| 80 | Wang et al. 2022 [97] | 62 | F | L4-5 | *C. tropicalis* | 1 | 30 | Flucytosine and amphotericin B | Laminectomy and pseudo-cystectomy | Fungal spondylodiscitis, secondary lumbar spinal stenosis, and subsequent discal pseudocyst causing acute cauda equina syndrome | A |
| 81 | Overgaauw et al. 2020 [98] | 78 | M | L4-5 | *C. krusei* | 1 | 6 | Anidulafungin and voriconazole | | Spondylitis | A |
| 82 | Supreeth et al. 2020 [99] | 50 | M | L4-5 | *C. auris* | 1 | 6 | Caspofungin | Posterior decompression, debridement, and stabilization | Spondylodiscitis | A |
